# Flexible Conductive Paper-Based Sensors for On-Skin Electrophysiological Monitoring and Wearable Applications

**DOI:** 10.1021/acsami.5c22432

**Published:** 2025-12-31

**Authors:** George Al Boustani, Lukas Bichlmaier, Tetsuhiko F. Teshima, Oleksandr Berezin, Lennart JK Weiß, Koji Sakai, Kenji Kondo, Lukas Hiendlmeier, Defne Tüzün, Beatrice De Chiara, Marta Nikić, Gil G Westmeyer, Shigeyoshi Inoue, Markus Becherer, Bernhard Wolfrum

**Affiliations:** † Neuroelectronics, Munich Institute of Biomedical Engineering, Department of Electrical Engineering, TUM School of Computation, Information and Technology, 9184 Technical University of Munich, Garching 85748, Germany; ‡ Medical & Health Informatics Laboratories, NTT Research Incorporated, Sunnyvale, California 94085, United States; § Chair of Silicon Chemistry, Department of Chemistry, Institute of Silicon Chemistry and Catalysis Research Center, TUM School of Natural Sciences, Technical University of Munich, Garching 85748, Germany; ∥ Faculty of Science and Technology, Keio University, Yokohama, 108-0073 Kanagawa 223−8522, Japan; ⊥ Munich Institute of Biomedical Engineering, TUM School of Natural Sciences & TUM School of Medicine and Health, Neurobiological Engineering, Garching 85748, Germany; # NTT Basic Research Laboratories and Bio-Medical Informatics Research Center, NTT Corporation, 3-1, Morinosato Wakamiya, Atsugi, Kanagawa 243-0198, Japan; ¶ New Materials Development Team, Wet-Jet Mill Technical Department, Plant Equipment Division, Sugino Machine Limited, 2880 Kuriyama, Namerikawa, Toyama 936-8577, Japan; ∇ Chip-Based Magnetic Sensor Technology, ZEITlab TUM School of Computation, Information and Technology, Technical University of Munich, 85748 Garching, Germany

**Keywords:** cellulose nanofibers (CNF), PEDOT:PSS, ionic liquids, conductive composites, flexible electrodes, wearables

## Abstract

Flexible, skin-conformable electrodes require materials that combine mechanical robustness, environmental stability, high electrical performance, and biocompatibility. Here, we present a flexible conductive composite film composed of poly­(3,4-ethylenedioxythiophene):polystyrenesulfonate (PEDOT:PSS), cellulose nanofibers (CNF), and the ionic liquid 1-ethyl-3-methylimidazolium ethyl sulfate (EMIM ES). The composite is fabricated via a simple aqueous blending and filtration process, yielding a free-standing film with a robust fibrous microstructure. ATR-FTIR analysis confirms the successful integration of all components, while SEM imaging reveals a percolated nanofibrillar architecture that enhances interfacial adhesion and structural integrity. Mechanical testing reveals a tensile strength of up to 335 MPa, accompanied by a strain of 21%, attributed to the increasing CNF content. Composite films with low CNF content exhibit excellent electrical stability across humidity levels between 10% and 90% and temperatures of 15–55 °C, and maintain electrochemical performance after 100,000 cycles of mechanical fatigue testing. On-skin electrophysiological recordings from a rodent model demonstrate stable signal acquisition without skin irritation, establishing the hybrid films as a promising platform for soft, wearable bioelectronic interfaces.

## Graphical abstract

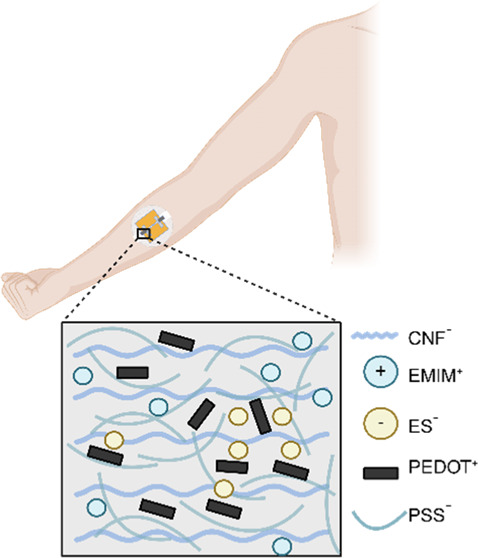

## Introduction

Flexible, skin-conformable electrodes are important for emerging applications in noninvasive medical diagnostics, health monitoring, and human–machine interfaces.^1–6^ These technologies require conductive materials that maintain electrical performance under complex mechanical deformations and across varying physiological environments.^5,7,8^ Although traditional thin-film metal electrodes offer high conductivity, their brittleness and rigidity render them unsuitable for long-term integration.^9^ To overcome these limitations, conductive polymers, such as poly­(3,4-ethylenedioxythiophene):polystyrenesulfonate (PEDOT:PSS), have gained attention due to their intrinsic organic composition, ionic conductivity, and favorable skin-interface properties.^9–15^

However, PEDOT:PSS, in its pristine form, remains mechanically brittle, particularly under repeated strain when fabricated as standalone films.^16,17^ Strategies to improve its mechanical resilience often involve incorporating secondary components such as dopants (e.g., glycerol, polytetrafluoroethylene) or blending with elastomers, hydrogels, and nanofibers.^18–22^ While such approaches can enhance mechanical properties and interface wetting, they often compromise conductivity or result in poor environmental durability, especially under fluctuating humidity and temperature conditions.^23^

To address these challenges, researchers have explored hydrogel-based or porous fibrous composites that embed within the PEDOT:PSS matrices.^18,24–28^ Such approaches enhance the mechanical characteristics of the film but also affect its electrical properties. One strategy that aims to improve the electrical performance is adding ionic liquids.^12,20,29^ These approaches offer improved electrical properties, but typically suffer from low electrical conductivity and mechanical stability under environmental stress.^30^ Achieving a balance between mechanical flexibility and electrical conductivity remains a key materials design challenge in this domain.^31^

Several studies have combined PEDOT:PSS with natural polymers such as cellulose nanofibrils, bacterial cellulose, and hydrogel matrices to create soft, conductive films for epidermal and implantable bioelectronics. These hybrid systems typically exhibit electrical conductivities in the range of 10–600 S cm^–1^, conformal adhesion to skin or tissue, and stable recording of electrophysiological signals.^32–35^ Despite these advances, significant challenges remain, including long-term stability under varying humid conditions, where ionic additives can leach and conductivity deteriorates.^36^ Moreover, low mechanical durability during repeated deformation may lead to delamination or crack formation under strain.^33,36^ In addition, manufacturing scalability is often challenging because many reported materials rely on multistep solvent exchange that are difficult to translate to large-area or continuous production.

Cellulose nanofibers (CNFs) serve as a robust structural matrix within such composites due to their exceptional mechanical strength and high surface area. CNFs provide mechanical reinforcement through a dense hydrogen-bonded network, yielding stiffness and tensile strength comparable to synthetic reinforcing fibers, while maintaining flexibility and toughness under cyclic strain.^37–40^ Their hydroxyl-rich surfaces facilitate strong hydrogen bonding and electrostatic interactions with PEDOT:PSS, enabling homogeneous polymer adsorption and the formation of uniform percolation networks that enhance charge carrier transport.^41,42^ Such interfacial coupling promotes mechanical integrity and conductivity retention under deformation. Additionally, CNFs enable scalable aqueous processing via vacuum filtration or drop casting, allowing uniform film formation with controllable porosity and thickness. Importantly, CNFs are renewable, biocompatible, and environmentally stable, aligning with sustainable design principles and long-term skin compatibility for wearable bioelectronics^43^

In this study, we report a composite strategy that synergistically integrates PEDOT:PSS, CNF, and an ionic liquid (IL)­1-ethyl-3-methylimidazolium ethyl sulfate (EMIM ES) to overcome the aforementioned challenges. CNFs are a robust, biocompatible scaffold with high mechanical strength, nanoscale porosity, and ease of processing via simple filtration. Their fibrous network facilitates mechanical reinforcement while maintaining flexibility. Meanwhile, the ionic liquid acts as a dopant and a charge transport mediator, improving electrical conductivity, flexibility, and thermal-moisture stability without compromising biocompatibility.^31^ Furthermore, the IL can be trapped through the filtration process due to the molecular interaction between the PEDOT:PSS and EMIM ES. Although binary systems combining PEDOT:PSS with ILs or CNFs have been reported individually, a ternary hybrid system that couples all three components, PEDOT:PSS, CNF, and IL, remains underexplored.

Herein, we develop and characterize a PEDOT:PSS-CNF-IL hybrid composite film fabricated via a facile aqueous filtration process, and evaluate its mechanical, electrical, environmental, and bioelectronic performance.^44^ We demonstrate that the composite achieves a favorable balance between electrical conductivity and mechanical compliance, exhibiting outstanding stability under cyclic fatigue, aging, and variations in temperature and humidity. Remarkably, it preserves its conductivity during and after exposure to moisture. The material’s utility is further validated as a skin-interfaced electrode for electrophysiological signal acquisition. This work thus establishes a scalable and biocompatible strategy for the fabrication of flexible electrodes in wearable bioelectronics. The resulting free-standing films can be precisely patterned by laser ablation into diverse sensor architectures and geometries tailored for specific applications.

## Results and Discussion

### Chemical Characterization

Attenuated total reflectance Fourier transform infrared (ATR-FTIR) spectroscopy was employed to investigate the molecular structures and confirm the composition of the individual components—CNF, EMIM ES, PEDOT:PSS, and the composite films. Spectra were recorded over the range of 4000–700 cm^–1^, and the characteristic absorption bands were interpreted to assess chemical integrity and potential interactions within the films. The ATR-FTIR spectrum of cellulose displayed a broad absorption band centered around 3320 cm^–1^ (Figure 1A), corresponding to the O–H stretching vibrations of intramolecular and intermolecular hydrogen bonds, and a C–O–C at around 1020 cm^–1^ consistent with its polysaccharide backbone (Figure 1B).^45,46^ The C–H stretching vibration of the imidazolium ring in EMIM ES can be seen between 2950 cm^–1^ and 3160 cm^–1^, while the signal at 1566 cm^–1^ can be attributed to the C=N stretching (Figure 1A).^47,48^ Additional bands observed in the 1300 cm^–1^ to 700 cm^–1^ region were attributed to symmetric and asymmetric S=O stretching modes of the ethyl sulfate anion.^49^ For PEDOT:PSS, the ATR-FTIR spectrum revealed distinct vibrational features including CH_2_ stretching near 2919 cm^–1^, C=C stretching at around 1550 cm^–1^, and a series of peaks in the 1300–700 cm^–1^ region corresponding to S=O stretching,^50^ C–O–C bending, and C–S–C skeletal vibrations (Figure 1A),^51^ indicative of both the 3,4-ethylenedioxythiophene and sulfonate moieties. The composite material low (L-) content of CNF (C) with PEDOT:PSS (P) and EMIM ES (E) film (L-CPE) displayed a superimposed spectral profile incorporating all major bands from the individual components, validating the successful integration of cellulose, EMIM ES, and PEDOT:PSS within the matrix. The presence of unaltered vibrational features in the L-CPE spectrum suggests that the chemical functionalities of each constituent were largely preserved during synthesis (the medium content (M-) of CNF, PEDOT:PSS, and EMIM ES film (M-CPE) and high content (H−) of CNF, PEDOT:PSS, and EMIM ES film (H–CPE) ATR-FTIR exhibited a similar spectrum as L-CPE (Figure S1. Additionally, minor shifts in peak positions and variations in band intensities are indicative of noncovalent interactions, such as hydrogen bonding or electrostatic forces, which support the formation of a physically integrated and chemically stable composite network.

**Figure 1:**
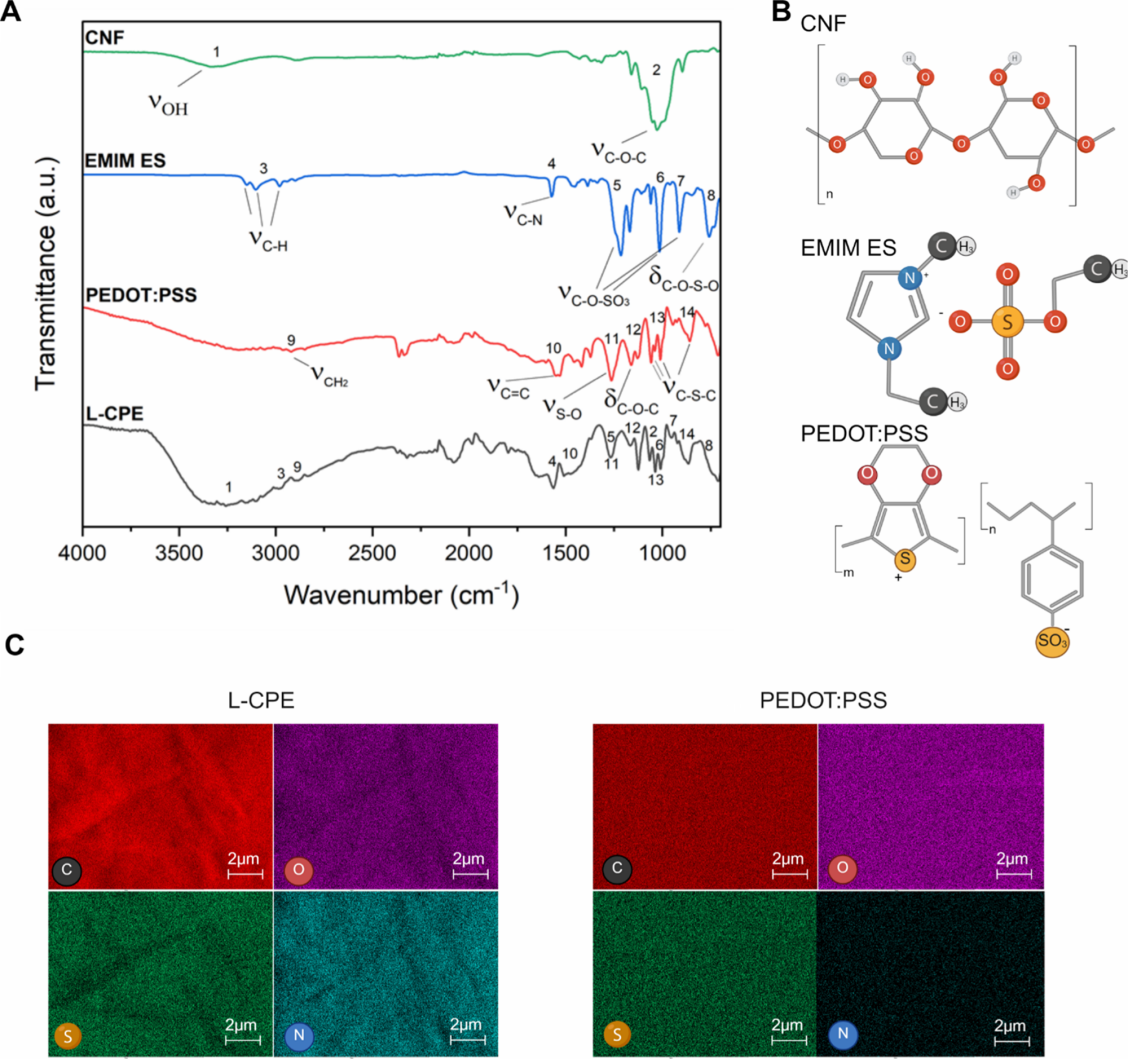
(A) ATR-FTIR spectra of CNF, EMIM ES, PEDOT:PSS, and the synthesized composite L-CPE, highlighting key vibrational modes. CNF exhibits broad O–H stretching (3320 cm^–1^) and C–O–C stretching (1020 cm^–1^). EMIM ES shows C–H (2950 cm^–1^ and 3160 cm^–1^) and C=N (∼1566 cm^–1^) stretching along with characteristic S=O vibrations (∼1200–700 cm^–1^). PEDOT:PSS displays CH_2_ (∼2919 cm^–1^), C=C (∼1550 cm^–1^), and skeletal vibrations (S–O, C–O–C, C–S–C) in the 1200–1000 cm^–1^ region. The L-CPE spectrum incorporates all major features, indicating the successful combination of the three components. (B) Molecular structures of CNF, EMIM ES, and PEDOT:PSS used in the composite preparation. (C) EDX elemental mapping of L-CPE and PEDOT:PSS surfaces.

Furthermore, L-, M-, and H–CPE were investigated via Raman spectroscopy (Figure S2). Characteristic Raman bands at 1249, 1367, 1433, 1504, and 1538 cm^–1^ can be attributed to vibrational modes of PEDOT, whereas the peaks at 990 and 1562 cm^–1^ originate from the polystyrenesulfonate.^52^ Compared to reference samples of pristine PEDOT:PSS and PEDOT:PSS and EMIM ES fim (PE), the signal sharpness and positions remain largely unchanged. The sharpness and positions of these signals are consistent with those of pristine PEDOT:PSS, indicating that the semicrystallinity and overall domain structure are mostly preserved upon incorporation of EMIM ES and CNF. In particular, the C=C stretching mode at 1433 cm^–1^ reflects the ratio between benzoid and quinoid resonance structures within the PEDOT backbone.^53,54^ The persistence of this distinct feature suggests a homogeneous distribution of these structures throughout the composite. Notably, a minor attenuation of the band at ∼1538 cm^–1^, accompanied by an increase in the 1504 cm^–1^ signal from pristine PEDOT to L-CPE, M-CPE, and H–CPE, points to a progressive transition toward a more planar and quinoid-like conformation of the PEDOT chains, indicating enhanced planarization of the conductive polymer domains with increasing EMIM ES and CNF content.^55^

Scanning electron microscopy (SEM) coupled with energy dispersive X-ray spectroscopy (EDX) elemental mapping was conducted to investigate the surface morphology and elemental distribution of the composite L-CPE compared to a control sample PEDOT:PSS. Elemental mapping of the L-CPE surface revealed a uniform distribution of key elements carbon (C), oxygen (O), sulfur (S), and nitrogen (N), which corroborates the successful incorporation of all major constituents: CNF, EMIM ES, and PEDOT:PSS (Figure 1C). Specifically, the presence of nitrogen is indicative of the imidazolium-based ionic liquid, confirming its homogeneous dispersion within the film matrix. The oxygen and carbon maps support the cellulose backbone and PEDOT:PSS, while the presence of sulfur-containing moieties further validates the integration of sulfonate groups from both PEDOT:PSS and EMIM ES. In contrast, the PEDOT:PSS sample lacked detectable nitrogen, confirming that the detection of nitrogen in L-CPE film is related to the EMIM ES (Figure 1C). Collectively, these results support the formation of an elementally homogeneous composite in L-CPE, with the efficient distribution of both organic and ionic functionalities across the material surface. Additionally, the EMIM ES was not filtered out during the vacuum filtering process.

### Mechanical Characterization

The surface morphology of the individual and composite films was examined using scanning electron microscopy (SEM) at both low and high magnifications to emphasize structural differences arising from the composite formulation. The pristine PEDOT:PSS film (Figure 2A) exhibited a smooth and homogeneous surface, with the high-magnification inset revealing discrete spherical domains that may be attributed to microscale phase segregation during film formation. In contrast, the PEDOT:PSS and EMIM ES film (PE) (Figure 2B) exhibited a less uniform topology, characterized by the presence of microscale protrusions and void-like features, likely due to residual stresses arising from drying or the gelatinization of the mixture. Notably, the morphology of the L-CPE film (Figure 2C), prepared by combining cellulose nanofibers (CNF) with PEDOT:PSS and EMIM ES, was significantly altered. The L-CPE surface exhibited a fibrous microstructure, indicating the successful incorporation and distribution of CNF within the conducting polymer matrix. These entangled fibrils enhance surface roughness and provide mechanical reinforcement. The M-CPE film (Figure 2D), which had a higher CNF concentration than L-CPE, showed a dense, pronounced fibrillar network. At high magnification, M-CPE revealed multiple-sized interconnected CNFs. The interconnected CNF structure is also evident in the CNF-only film (Figure S3). The fibrous structure observed in both L-CPE and M-CPE contrasts sharply with the amorphous or phase-separated morphologies of PEDOT:PSS and PE, underscoring the role of CNF in templating and structuring the composite architecture.^56^

**Figure 2:**
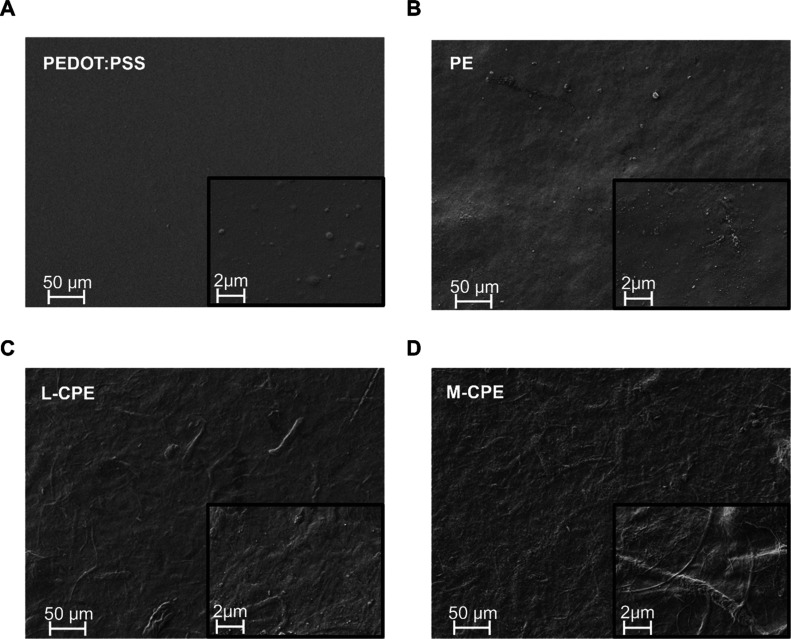
(A) SEM micrographs of surface morphologies at low (main image) and high magnification (insets) for (A) PEDOT:PSS, (B) PE, (C) L-CPE, and (D) M-CPE films. PEDOT:PSS shows a smooth, featureless surface with dispersed spherical domains likely resulting from phase separation. PE exhibits a rougher surface with microscale imperfections but lacks fibrous features. In contrast, L-CPE and M-CPE both display fibrous, network-like morphologies indicative of the integration of cellulose nanofibers. The higher density and interconnectivity of fibrils in M-CPE compared to L-CPE suggest enhanced structural reinforcement and polymer dispersion, contributing to improved mechanical and electrical properties in the composite films. The SEM image was made using a secondary electron detector and an acceleration voltage of 2 kV.

The mechanical properties of the composite films were evaluated through tensile testing, and the resulting stress-strain curves are presented in Figure 3A. The PE film exhibited low tensile strength and strain at break (end of curve), indicative of its brittle nature and limited mechanical reinforcement. In contrast, all CNF-reinforced composite samples L-CPE, M-CPE, and H–CPE demonstrated significant improvements in both maximum stress and strain, highlighting the role of cellulose nanofibers (CNF) in enhancing the mechanical integrity of the polymer matrix (Figure 3B). Notably, the tensile strength increased progressively with CNF content, as indicated by the trend from L-CPE to H–CPE. The measured tensile strengths were 32, 71, 170, and 335 MPa for PE, L-CPE, M-CPE, and H–CPE, respectively. The strains at the maximum elongation at break were 12%, 24%, 21%, and 21%, suggesting load-bearing capacity and ductility for the nanofiber-containing materials. This enhancement can be attributed to the reinforcing effect of the CNF network, which provides both structural rigidity and flexibility through efficient stress transfer and energy dissipation during deformation. The increased interfacial interaction between CNF and the conducting polymer matrix likely contributes to better mechanical coupling, enabling the composite to withstand higher stress than PE. These findings underscore the effectiveness of CNF as a filler for improving strength and toughness of conductive polymer-based flexible films.

**Figure 3:**
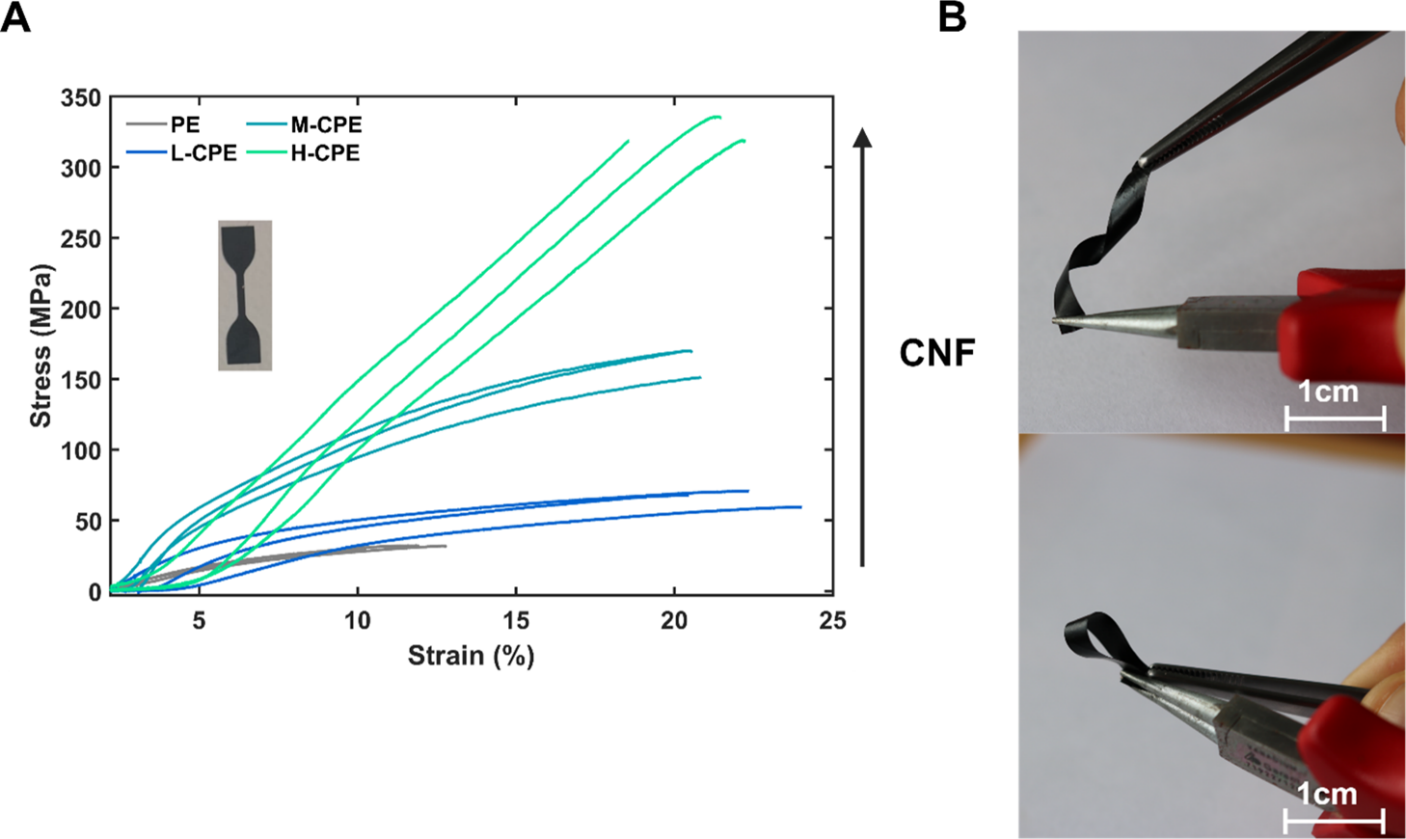
(A) Stress-strain curves of PE, L-CPE, M-CPE, and H–CPE films measured via tensile testing until rupture. The PE film shows poor mechanical performance with low tensile strength and elongation. CNF-reinforced composites (L-CPE, M-CPE, and H–CPE) exhibit significant improvements in both stress and strain, with mechanical properties progressively enhanced with increasing CNF content. The H–CPE film demonstrates the highest tensile strength of 335 MPa and strain of 21%, confirming the reinforcing and toughening effect of cellulose nanofibers within the composite matrix. (B) L-CPE images showing the flexibility of the films.

### Electrical Characterization

Electrical characterization was conducted on the developed materials, focusing on their resistance values and changes under controlled experimental conditions. The electrical characterization involved several techniques, including sheet resistance measurements utilizing a direct current four-probe method at laboratory temperature and relative humidity (RH), which assesses the material’s electrical properties by minimizing contact resistance effects. Moreover, the sheet resistance measurements were performed at different temperatures and relative humidity levels to assess the impact of environmental conditions on the material’s electrical performance. Then, impedance spectroscopy was conducted to analyze the material’s impedance within a range of frequencies. Finally, a cyclic fatigue test (axial tension and compression) was conducted to evaluate the durability and stability of the materials over time, providing insight into their long-term reliability.

Comparing the electrical properties of the various materials, the PE sheets and the L-CPE sheets exhibit the lowest sheet resistance values of 0.90 ± 0.18 Ω/sq and 0.79 ± 0.14 Ω/sq, respectively, as well as resistivity values, with PE measuring 5.15 × 10^–5^ Ωm and L-CPE 6.09 × 10^–5^ Ωm (Table 1). The low sheet resistance observed in the materials is primarily attributed to the interactions between PEDOT:PSS and EMIM ES, as well as the low amount of CNF in the case of L-CPE see Table S1. Pristine PEDOT:PSS films exhibit a sheet resistance of 1.43 ± 0.29 Ω/sq. The ionic interactions between the ionic liquid and the conductive polymer generate pathways for charge carriers, reducing resistance and enhancing overall electrical performance. Such interactions can lead to increased charge mobility and network connectivity, lowering the sheet resistance of the materials. In contrast, the M-CPE and H–CPE sheets show an increase in sheet resistance to 4.85 ± 3.24 Ω/sq and 28.9 ± 8.30 Ω/sq (Table 1). The higher sheet resistance values and higher standard deviation are attributed to the increased percentage of CNF in the sheet compositions, with 50% and 90% of CNF, respectively.

**Table 1:** Electrical Characteristics of Various Materials^a^

material (*N* = 10)	sheet resistance (Ω/sq) (22 °C, 60% RH)	thickness (μm)	resistivity (Ωm) (22 °C, 60% RH)
PE	0.90 ± 0.18	57.2 ± 8.58	5.15 × 10^–5^
L-CPE	0.79 ± 0.14	77.1 ± 2.28	6.09 × 10^–5^
M-CPE	4.85 ± 3.24	46.4 ± 4.36	2.25 × 10^–4^
H–CPE	28.9 ± 8.30	54.2 ± 8.98	1.60 × 10^–3^
CP	643 ± 541	41.4 ± 4.13	2.66 × 10^–2^
CE	1.01 × 10^7^ ± 9.88 × 10^6^	12.5 ± 3.07	1.26 × 10^2^

a pedot:pss (p), cnf (c), and 1-ethyl-3-methylimidazolium ethyl sulfate (e) used in the preparation of composite films. l-cpe, m-cpe, and h–cpe represent low-, medium-, and high-cellulose-content formulations, respectively. the resistivity was calculated by dividing the average sheet resistance with the average thickness of each film.

The CP and CE films demonstrate even higher sheet resistance and resistivity values, particularly CE, which has a sheet resistance of 1.01 × 10^7^ ± 9.88 × 10^6^ Ω/sq. The CP and CE films serve as reference materials, where the CP films do not contain any EMIM ES, and the CE films do not contain any PEDOT:PSS. Hence, the lower observed sheet resistance in PE, L-CPE, M-CPE, and H–CPE can be attributed to the interaction between EMIM ES and PEDOT:PSS. A summary of the electrical and mechanical properties is provided in Table S2.

To evaluate the environmental robustness of the composite films for potential use in flexible and wearable electronics, electrical stability was systematically investigated under varying temperature and humidity conditions. The sheet resistances of PE, L-CPE, and M-CPE films were measured across a temperature range of 15–55 °C at a constant 80% RH (Figure 4A). PE samples exhibited a slight drift in the sheet resistance with values ranging from 0.69 ± 0.14 Ω/sq at 15 °C to 0.82 ± 0.16 Ω/sq at 55 °C. However, L-CPE samples exhibited stable and low sheet resistance values, ranging approximately from 0.70 ± 0.02 Ω/sq at 15 °C to 0.73 ± 0.02 Ω/sq at 55 °C, with minimal fluctuation across the temperature sweep, indicating thermally stable charge transport. In contrast, M-CPE maintained a consistent sheet resistance between 1.63 Ω/sq and 1.64 Ω/sq across all temperatures, suggesting a more resistive microstructure to temperature due to the higher amount of CNF. Similarly, humidity-dependent measurements conducted at a constant temperature of 40 °C (Figures 4B and S4) showed negligible change in relative humidity values for L-CPE and PE across a wide relative humidity range (30% RH to 90% RH), further emphasizing the composites’ insensitivity to moisture exposure. However, the M-CPE sheet resistance values decreased from 1.72 ± 0.3 Ω/sq at 30% RH to 1.67 ± 0.29 Ω/sq at 90% RH.

**Figure 4:**
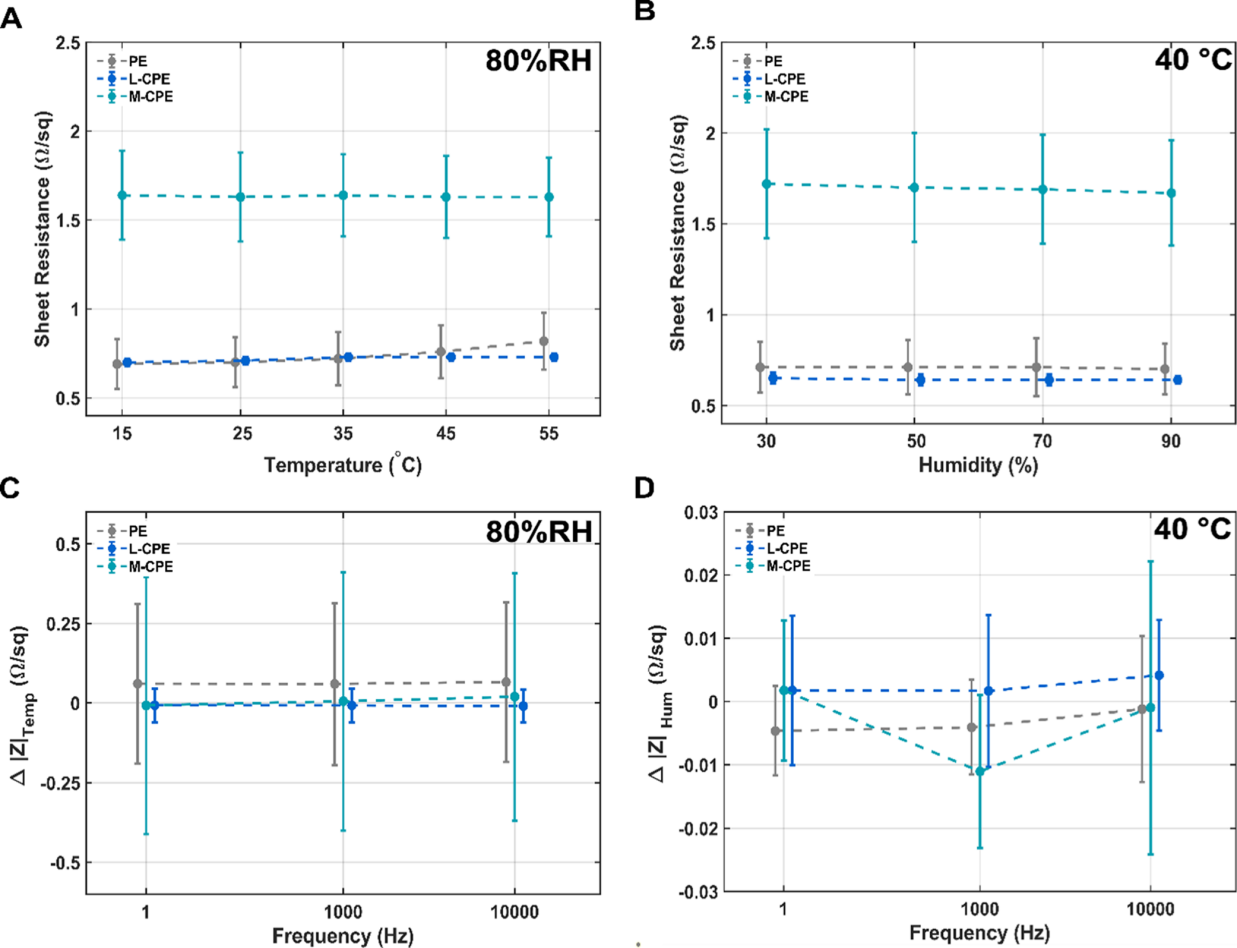
Environmental stability of PE, L-CPE, and M-CPE films with respect to temperature and humidity, evaluated through sheet resistance and impedance measurements. (A) Temperature-dependent sheet resistance at 80% RH across 15 to 55 °C. (B) Humidity-dependent sheet resistance at 40 °C across 30% RH to 90% RH relative humidity. (C) Frequency-dependent difference in impedance between 15 and 55 °C at 80% RH. (D) Frequency-dependent difference in impedance between 30% RH and 90% RH relative humidity at 40 °C. L-CPE and PE films exhibit stable electrical behavior across different environmental conditions, while M-CPE shows slightly higher variability attributed to structural or compositional differences. Data represent mean ± SD (*N* = 3). The average sheet resistance measurements shown in (A,B) deviate from each other and the reported values in Table 1 as they represent a subset of 3 samples.

Impedance spectroscopy was conducted to complement these findings by evaluating the response of the films to environmental stimuli across a wide frequency domain (1 Hz to 10 kHz). As shown in Figure 4C, the temperature-induced difference in impedance magnitude between 15 and 55 °C had small drifts across all frequencies for PE and L-CPE, confirming that thermal fluctuations did not strongly disrupt charge transport pathways. In contrast, M-CPE exhibited a slightly higher impedance difference (Δ|Z|), especially at lower frequencies, indicating possible thermal modulation of ionic motion or microstructural rearrangement. The humidity-dependent impedance changes at 40 °C (Figure 4D) were minimal for all materials, with L-CPE showing the most stable curves. Furthermore, all three sheets PE, L-CPE and M-CPE demonstrated a constant impedance between 0.1 Hz and 10 kHz (Figure S5C). The constant impedance in combination with an approximately zero phase across the measured frequencies suggests that the three tested sheets are almost purely resistive between 0.1 Hz and 10 kHz (Figure S5C).

Overall, the L-CPE composite demonstrates a balance of conductivity and environmental stability, maintaining its electrical performance under both temperature and humidity variations. This robust behavior is likely attributed to the synergistic interaction between the conductive polymer matrix and the mechanically reinforcing, hydrophilic cellulose nanofiber network, which stabilizes the morphology and conductive pathways together. These findings support the suitability of L-CPE composites for applications requiring consistent electrical functionality under varying environmental conditions, such as bioelectronic interfaces, wearable sensors, and soft energy devices.

The impedance drift was measured at 1 kHz during continuous exposure to artificial sweat for over 24 h. We observed only a minor drift of 2% change over the entire testing period (Figure 5A). The aging test was employed to evaluate the long-term electrical stability of the conductive films by exposing them to constant environmental conditions, including humidity and temperature, over time. This experiment quantified the absolute relative change in sheet resistance of PEDOT:PSS (P), and L-CPE films after a 15 day passive aging period under constant temperature and humidity (*N* = 3). The pristine PEDOT:PSS (P) films exhibited a pronounced increase in sheet resistance, accompanied by substantial variability, indicating severe electrical degradation and poor stability (Figure 5B). Further improvement was observed for L- CPE films, which combine PEDOT:PSS with both EMIM ES and CNF, yielding even smaller absolute relative change of resistance value and enhanced resistance to aging.

**Figure 5:**
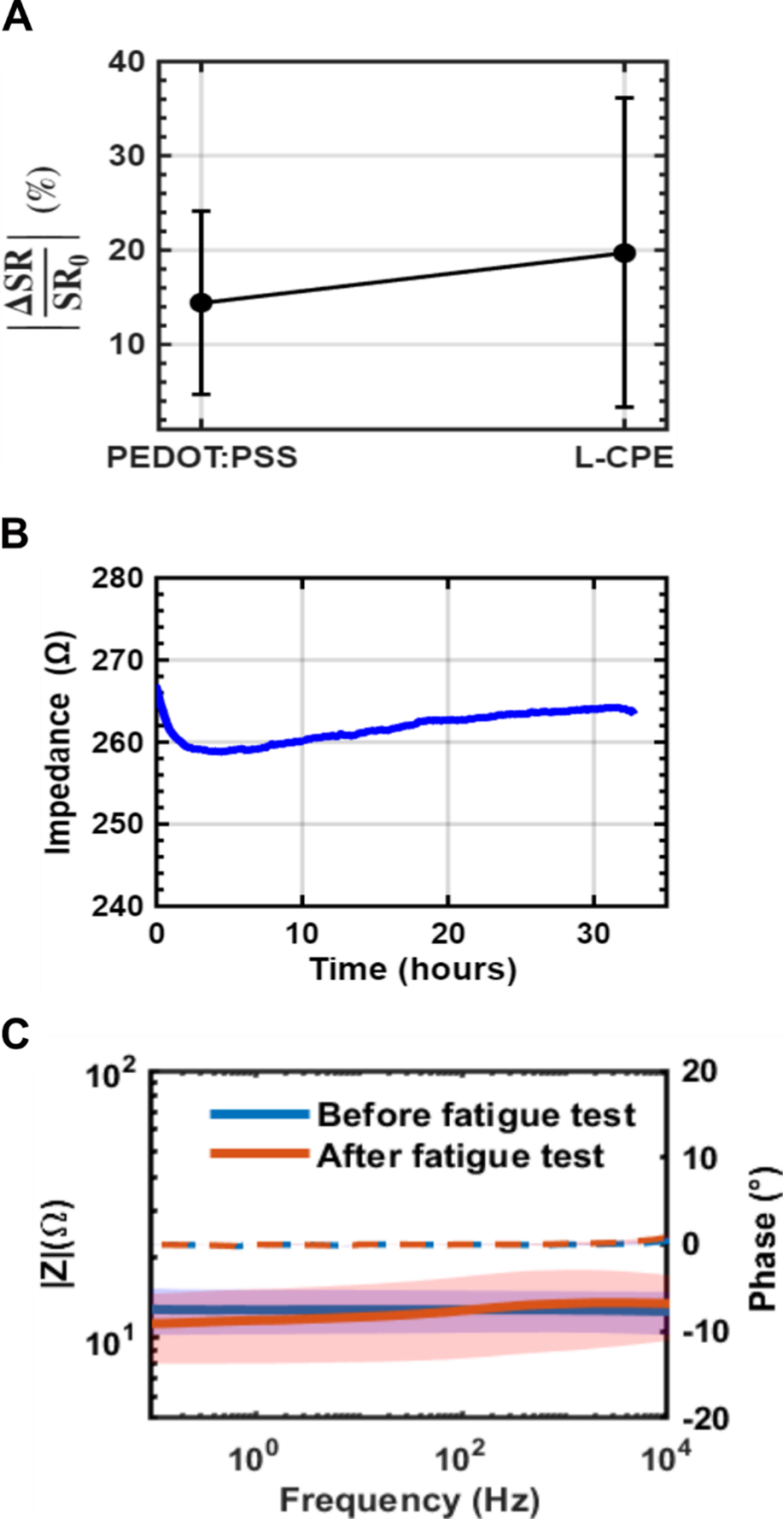
(A) Aging tests were performed at 80% RH, 35 °C for 15 days. The plot shows the absolute relative difference of the sheet resistance before and after the aging test for PEDOT:PSS (P), and L-CPE, (*N* = 3). (B) The impedance of an L-CPE electrode was measured at a fixed frequency of 1 kHz under continuous exposure to artificial sweat for over 24 h. (C) Impedance spectroscopy results of L-CPE composite films in dry conditions at room temperature before and after 100,000 cycles of mechanical fatigue testing. Bode plot showing the impedance magnitude (|*Z*|) as a function of frequency (logarithmic scale) before the fatigue test (blue) and after the fatigue test (red). The corresponding phase angles before and after the fatigue test are shown as dashed blue and dashed red lines, respectively. Minimal changes in |*Z*| and a phase angle close to 0° indicate that the electrical resistive behavior and structural integrity are preserved after mechanical deformation, demonstrating the composite’s robustness for flexible and fatigue-prone device applications.

An impedance spectroscopy was performed before and after a cyclic fatigue test to assess the durability of the L-CPE composite under repeated mechanical deformation. Prior to fatigue testing, the impedance profile showed characteristic features of a conductive material: a relatively low and stable |*Z*| value across the frequency range. After 100,000 cycles of mechanical loading (fatigue test), the impedance spectrum remained largely unchanged (Figure 5C). The slight reduction in |*Z*| values observed in the postfatigue response, especially in the low-frequency region, could indicate improved charge transport via stress-induced microrearrangements or better contact formation between conductive domains. The phase angle plot (Figure 5C) further supports this stability; no significant shift was observed across the frequency spectrum, indicating that the dielectric and conductive characteristics of the material were preserved after cyclic deformation. Together, these results confirm the electrochemical and structural resilience of the L-CPE composite, highlighting its potential for applications in dynamic or flexible electronic devices where long-term mechanical loading is expected.

### On-Skin Application

To evaluate the influence of residual ionic liquid on material cytotoxicity, we conducted a combined analysis using nuclear magnetic resonance (NMR) and a WST-8 cell viability assay in accordance with ISO 10993–5. The NMR spectrum of the residue collected after filtering the L-CPE formulation exhibited characteristic peaks corresponding to EMIM ES, indicating that traces of the ionic liquid remained bound within the solid matrix, while a portion of unbound EMIM ES was removed during filtration (Figure 6A). Following fabrication of the L-CPE film, minor but distinct resonance signals associated with EMIM ES were still observed in the 1 h NMR spectrum, confirming the presence of small amounts of nonencapsulated ionic liquid (Figure 6B). To further verify this observation, the films were immersed in distilled water for 1 min, air-dried, and reanalyzed in fresh D_2_O. No significant EMIM ES signals were detected after immersion of the washed L-CPE in D_2_O for 10 min, 12 h, and 24 h (Figure 6B), confirming that residual ionic liquid can be effectively removed by a simple washing step. These results demonstrate that the detected signals originated from surface-bound residues rather than ongoing leakage, and that the encapsulated ionic liquid remains stable within the L-CPE matrix.

**Figure 6:**
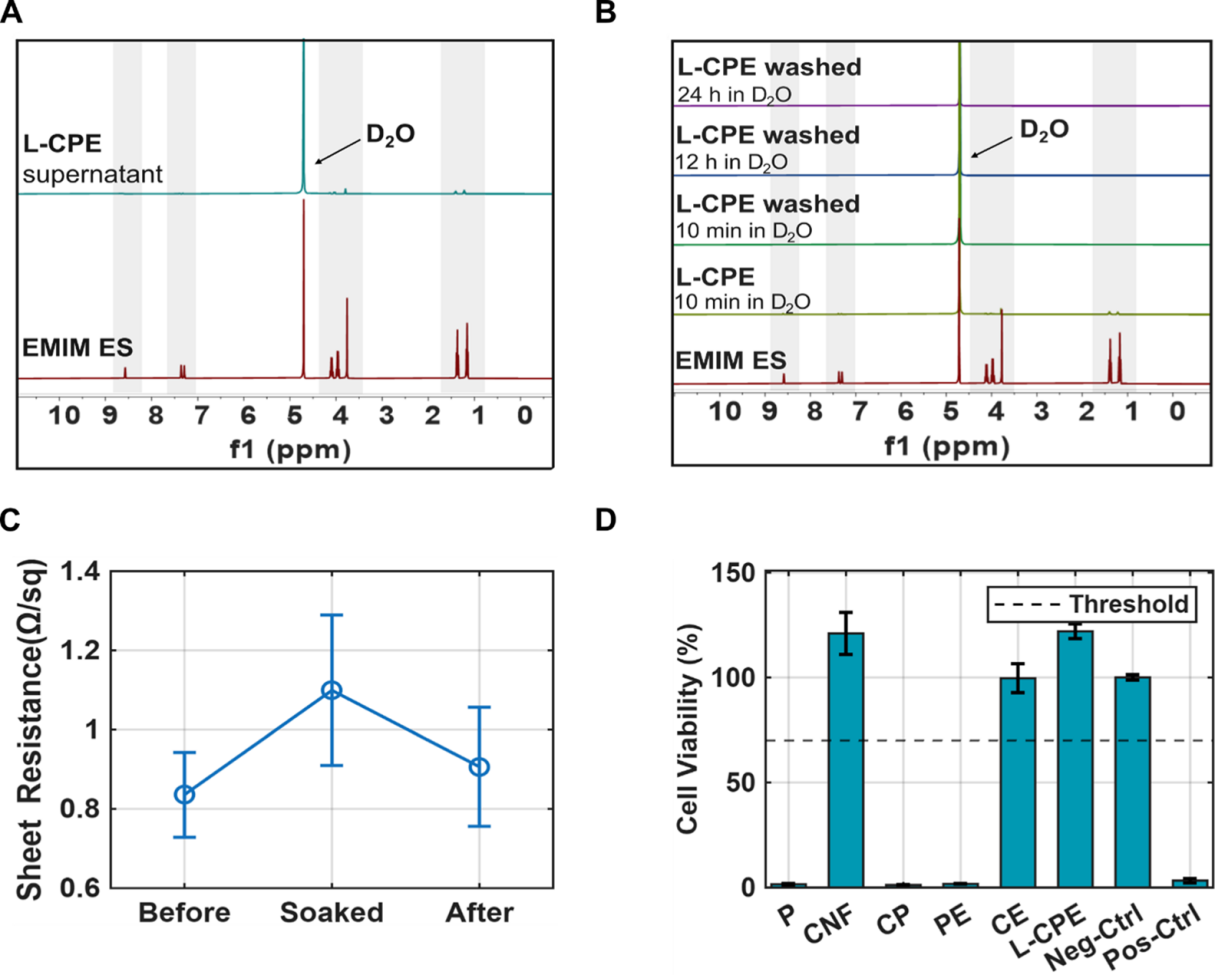
(A) NMR spectrum of the filtered residue from L-CPE showing characteristic peaks of residual EMIM ES, confirming partial release into the solution. (B) NMR spectra of L-CPE samples washed in D_2_O for increasing durations (10 min, 12 h, and 24 h), demonstrating progressive removal of EMIM ES over time. (C) Sheet resistance of the electrodes measured before, during PBS soaking, and after drying (*N* = 4). (D) Cell viability of various material formulations after 2 h of incubation, assessed using the WST-8 assay. Materials containing residual EMIM ES (e.g., PE, CP) show significant cytotoxicity, while washed L-CPE demonstrate noncytotoxic behavior, exceeding the 70% viability threshold (dashed line). Positive and negative controls validate the assay’s performance (*N* = 3).

The stability of the conductive network upon hydration was further assessed by measuring the sheet resistance of four L-CPE samples before and after immersion in phosphate-buffered saline (PBS) and subsequent drying. The sheet resistance increased from approximately 0.83 Ω/sq to 1.1 Ω/sq after PBS exposure and returned to 0.90 Ω/sq after drying (Figure 6C). This corresponds to a relative change of ∼8% between initial and final state, suggesting that hydration induces a temporary disruption of the conductive pathways, likely due to polymer swelling, while electrical performance is largely restored upon drying. The increased variability during the PBS-soaked state likely arises from differences in water uptake or microstructural rearrangement within the films.

To assess biocompatibility, a WST-8 cytotoxicity assay was performed following a 3 h incubation (Figure 6D). Several formulations, including PEDOT:PSS (P), CP, and PE, exhibited marked cytotoxicity, with cell viability well below the 70% threshold defined by ISO 10993–5. This indicates that unmodified or insufficiently purified materials pose cytotoxic risks, likely due to residual ionic liquid. In contrast, CNF-, CE-, and L-CPE-based films displayed cell viabilities exceeding 70%. The inclusion of appropriate positive and negative controls validated the assay, yielding expected high and low viability responses. Overall, these results confirm that proper formulation and purification, particularly the removal of unbound EMIM ES and the incorporation of stabilizing CNF, are crucial for ensuring cytocompatibility of these composites for bioelectronic applications.

Electrophysiological signal recording was performed using a rodent model to evaluate the practical bioelectronic applicability of the L-CPE composite films. As shown in Figure 7A, a flexible L-CPE-based electrode patch was conformally attached to the skin over the thoracic region of an anesthetized rat, with standard Ag/AgCl reference and ground electrodes placed on adjacent areas. The electrode patch-maintained contact with the skin, aided by its softness and conformability, which is essential for minimizing motion artifacts and ensuring signal stability during electrocardiography measurements. As shown in Figure 7B, the top trace demonstrates stable long-term raw signal acquisition over 5 min, highlighting the electrode’s low-noise interface and consistent skin-electrode contact. The middle panel displays a 10 s window after applying a high-pass filter,^57^ showing periodic and well-defined waveforms, likely corresponding to cardiac cycles. The bottom trace, with subsecond resolution, clearly resolves distinct waveform components, including P, QRS, T-like features (Figure 7B) with an average of 213 bpm and a signal-to-noise ratio of 20 dB (17 dB commercial Ag/AgCl benchmark) (Figure S6).^58^ The SNR was calculated by dividing the average RMS amplitude of the R–S waveforms by the RMS of the baseline noise­(13 a.u).^59^

**Figure 7:**
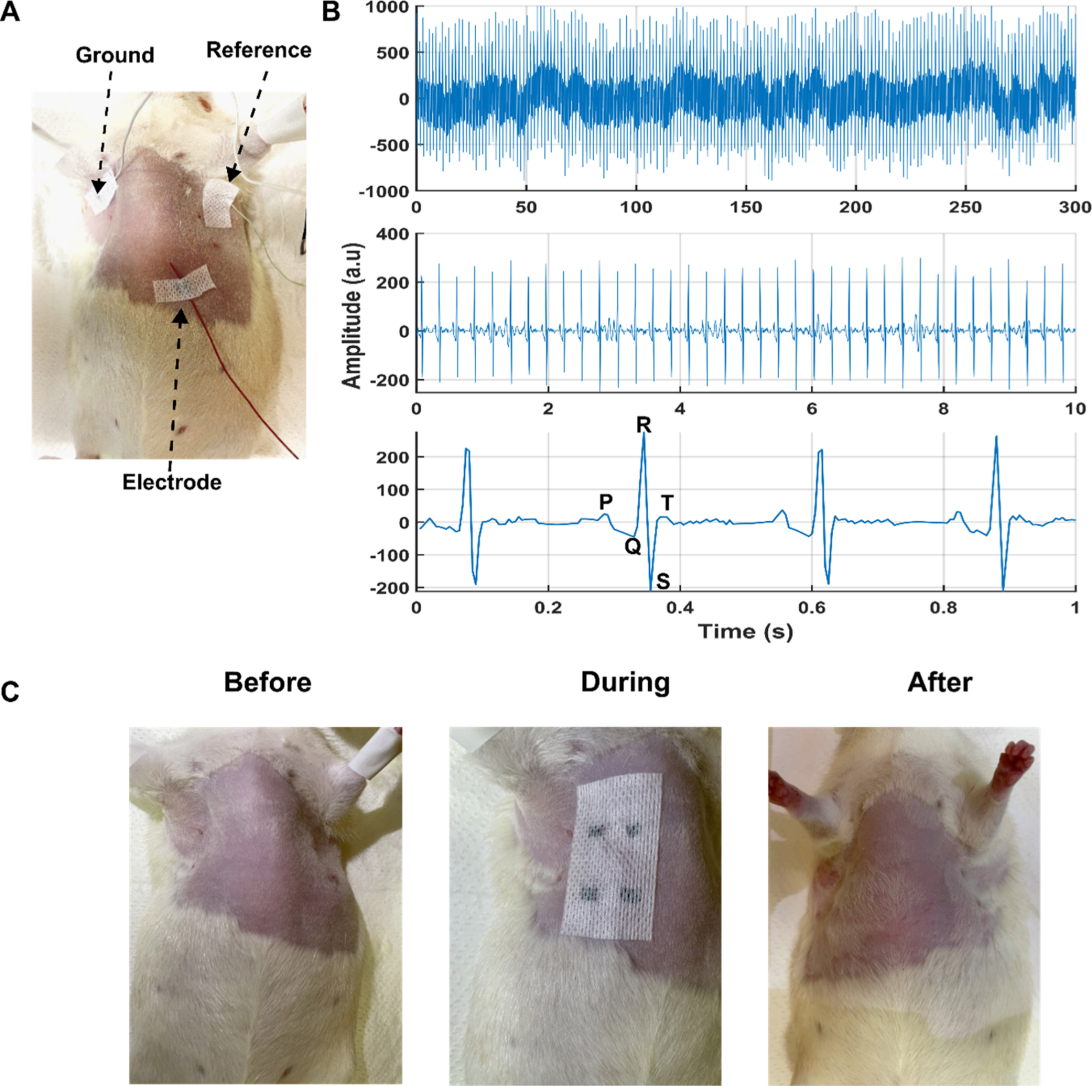
In-vivo evaluation of L-CPE composite electrodes for electrophysiological signal acquisition. (A) Optical image of the electrode setup on a rat model, with an L-CPE patch placed on the chest and standard reference/ground electrodes applied. (B) Real-time signal acquisition showing high-fidelity electrophysiological recordings over different time windows: full trace (0–300 s), intermediate (0–10 s), and zoomed-in view (0–1 s), with distinct waveform features observed. The measured data had a SNR of 20 dB. (C) Photographs of the skin region before, during, and after film application, confirming skin conformity and no observable irritation.

To assess the biocompatibility and skin tolerance of the device, photographs were taken before, during, and after the 1 week (Figure 7C). The skin appeared unaffected postremoval, with no signs of redness, irritation, or damage, indicating that the L-CPE patch is noninvasive and safe for skin-interfaced applications. Furthermore, an on-skin impedance spectroscopy measurement showed that the CPE films exhibited similar impedance as surface gold electrodes (Figures S8 and S9).^60^ These results validate the L-CPE composite as a promising candidate for bioelectronic interfaces, offering high-quality signal acquisition combined with skin-conformable wearability and biocompatibility.

## Conclusion

We have developed a flexible, conductive composite based on CNF, PEDOT:PSS, and the ionic liquid EMIM ES, and systematically evaluated its physicochemical, mechanical, and bioelectronic performance. ATR-FTIR analysis confirmed the successful incorporation of all components, with preserved functional groups indicative of a nondestructive fabrication method. Morphological analysis via SEM revealed that CNF introduces a fibrous and entangled microstructure, enhancing interfacial adhesion and dispersion of the conducting polymer matrix.

Mechanical testing demonstrated that CNF reinforcement significantly improves both tensile strength and elongation, validating the composite’s structural robustness. Environmental electrical stability was verified by monitoring sheet resistance and impedance across a broad temperature (15 to 55 °C) and humidity (30% RH to 90% RH) range, where L-CPE exhibited minimal variation, indicating reliable conductivity under diverse conditions. Electrochemical impedance spectroscopy performed before and after fatigue testing revealed a negligible change in impedance magnitude and phase, confirming electrical durability under mechanical cycling.

Bioelectronic measurements further demonstrated the material’s capability for real-time electrophysiological signal acquisition, with stable cardiac signals recorded in a rodent model. Taken together, these results highlight the potential of L-CPE as a conductive film for flexible and skin-conformable electronics, combining mechanical integrity, environmental and electrochemical stability, and good signal quality for physiological monitoring applications.

## Experimental Section

### Materials

The developed materials consist of three components: CNFs fibrillated using a water-jet homogenization (Sugino Machine Limited Japan), 1.3 wt % PEDOT:PSS (Clevios PH1000 from Heraeus Deutschland GmbH Germany) dispersion in water, and EMIM ES (> 98% purity, IoLiTec- Ionic Liquids Technologies GmbH Germany).

### Film Fabrication

First, the CNF mixture was formed by homogeneously sonicating 25 g of CNF with 475 g of deionized water for 40 min at 2 W with a 5 s on/off pulse (Figure S7A). The CNF and PEDOT:PSS were mixed, as shown in Table S1, and then magnetically stirred for 15 min. Finally, EMIM ES was added, and the solution was mixed by hand for 2 min (Figure S7B).

The fabrication method involves pipetting the prepared mixture into a vacuum filtration setup, as illustrated in Figure S7C. The setup consists of a 10 L vacuum motor connected to a filtration flask. A magnetic holder ensures a tight seal between the funnel and the porous plate, while a rubber bung secures the funnel to the flask. A polyvinylidene difluoride (PVDF) membrane filter (0.1 μm pore size, Durapore, Sigma-Aldrich GmbH) is placed on the porous plate. Prior to use, the PVDF membrane is rehydrated with deionized water, and the composite mixture is subsequently applied using a glass pipet.

All sheet formulations were prepared based on solid content, with 0.1 wt % for CNF and 1.1 wt % to 1.3 wt % for PEDOT:PSS. The ionic liquid EMIM ES was incorporated at a 1:10 volume ratio relative to the total volume of PEDOT:PSS and CNF. At higher ionic liquid contents, the filtering process became less reliable.

Consequently, five types of composite films were fabricated with the addition of EMIM ES: PE contained 100% PEDOT:PSS without any CNF; M-CPE consisted of 50% PEDOT: PSS and 50% CNF; and H–CPE contained 10% PEDOT:PSS and 90% CNF. CI contained 0% PEDOT:PSS and 100% CNF. Additionally, CP contained 50% PEDOT:PSS and 50% CNF without the addition of EMIM ES. This range of formulations enabled the investigation into the role of CNF content and ionic liquid incorporation on the composite films’ mechanical, electrical, and structural properties.

### Chemical Characterization

All samples were characterized by ATR-FTIR (Bruker Vertex 70v) with a wavenumber range of 4000 cm^–1^–700 cm^–1^ to assess the composition of the sheets after filtration. Raman spectra (inVia Reflex Raman System, Renishaw) of all composite formulations and pristine PEDOT:PSS were obtained using a 532 nm excitation laser with a grating of 1800 lines/mm. The laser power was set to 5% and the exposure time was 1 s. For NMR analysis, samples were prepared in 0.4 mL of D2O. For L-CPE, 0.4 mg of material was either directly dispersed in the NMR solvent or rinsed 60 s with H_2_O before drying and redispersion. NMR spectra were recorded using a Bruker Avance Neo 400 MHz spectrometer. The evaluation of the spectra was performed using MestReNova (version 15.0.0).

### SEM and EDX

The surface morphology and elemental composition of the film composites were characterized using SEM (Crossbeam 550 FIB-SEM, Carl Zeiss Microscopy AG Germany) coupled with energy-dispersive X-ray spectroscopy (Ultim Extreme windowless EDX detector, Oxford Instruments, United Kingdom). SEM imaging was performed to assess the microstructural features of the film surfaces with a primary electron beam acceleration voltage of 2 kV and a current of 200 pA, using a secondary electron detector for surface-sensitive signal detection. For the CNF sample, a primary electron beam energy of 2 kV and 500 pA current was used to generate an image with the SE2 detector. The EDX elemental mapping was employed to determine the spatial distribution of key elements (C, O, S, and N) across the samples. All EDX measurements were conducted using a primary electron beam energy at 5 kV and 200 pA beam current for characteristic X-ray energy spectrum excitation, keeping the SE2 collector bias voltage at 50 V for corresponding SE2 electron image generation. Sample drift correction was employed to ensure that EDX signals were collected from the same region.

### Tensile Testing

Tensile testing (Universal Testing Machines model 106, TesT GmbH, Germany) was performed to evaluate the mechanical properties of the composite films, including tensile strength, elongation at break, and overall ductility. Dumbbell-shaped structures from the films were fabricated using a laser cutter (Figure 3) (MD-U1000C, Keyence Japan) according to the ISO 527–3:2019–02 type 5 (downsized by a factor of 6) and mounted onto a tensile testing machine equipped with a 50 N load cell. Tests were carried out at room temperature under ambient humidity conditions using a constant extension rate of 5 mm·min^–1^ (*N* = 3). The stress–strain curves obtained from the measurements provided information on the influence of CNF on the mechanical behavior of the films.

### Fatigue Test

The fatigue test characterized the stability of the sample after multiple axial tension and compression cycles (5–6% strain and 80–90 deg bending cycle, ISO 13003:2003). The test is performed with a tensile tester and an impedance spectroscopy measurement using a PalmSens 4 electrochemical cell (PalmSens BV, The Netherlands). First, five samples were lasered into a dumbbell-shaped structure using a laser cutter. Impedance spectroscopy was performed between 0.1 Hz and 10 kHz at 10 mV amplitude. Then, the samples were clamped with the tensile tester and displaced inward by 1 cm 100,000 times. Finally, impedance spectroscopy was performed after the fatigue test with the same parameter range as before.

### Film Thickness Characterization

The thickness of the composite films was characterized using a laser microscopy profilometer (VK-X250, Keyence Japan) to ensure consistency and evaluate fabrication uniformity. Measurements were conducted at three distinct locations across the surface of each film to account for potential spatial variation and edge effects. The average thickness was calculated from these readings and reported as the mean ± standard deviation. This method provided noncontact, high-resolution profiling of the film surface, enabling precise assessment of thickness variation resulting from the vacuum filtration process.

### Electrical Characterization

The sheet resistance (SR) measurement was performed using a spring-loaded header probe, with a pin separation of 0.1 cm. The probe was attached to a micromanipulator using a 3D-printed holder (S3 Ultimaker BV, The Netherlands) made of acrylonitrile butadiene styrene (ABS). The four-probe measurement was performed using a VMP300 biologic system (VMP300, BioLogic, France) with stepwise chronoamperometry measurements. The voltage range was between −10 mV and 10 mV with a step of 2 mV. Furthermore, the sampling time was set to 0.1 s, with a 5 s measurement per step.

The temperature and humidity tests aim to characterize the relative resistance change of the material in response to changes in humidity and temperature. The tests were performed inside a climate chamber (Vötsch VCL4006, Vötsch Industrietechnik GmbH, Germany), and the sheet resistance was assessed using the four-probe measurement mentioned above. The temperature test was performed by maintaining a humidity level of 80% RH and increasing the temperature from 15 to 55 °C in increments of 10 °C. The humidity test was performed by fixing the temperature at 40 °C and varying the relative humidity between 30% RH and 90% RH in increments of 20% RH. Furthermore, in order to reach the desired surface temperature, a 40 min wait time was applied before measuring the sheet resistance upon reaching a certain temperature or humidity. Additionally, electrochemical impedance spectroscopy was performed between 1 Hz and 10 kHz (10 mV amplitude) for each temperature/humidity step. The measurement aimed to characterize potential drifts in impedance. The aging test was performed using the same climate chamber mentioned above at a temperature of 35 °C and a humidity of 80% RH for 15 days. The sheet resistance was measured before and after for PEDOT:PSS and L-CPE films (*N* = 3). A drift test was performed by lasering a sensor spot with a 3 mm diameter, and a feedline, 5 mm in length and 0.4 mm in width. The sensor was dipped inside an artificial sweat solution (pH 6.5, Synthetic Urine e.K., Germany). The impedance at 1 kHz was measured for more than 24 h (PalmSens4, PalmSens BV, The Netherlands) using an Ag/AgCl reference electrode.

### Cytotoxicity Test

The cytotoxicity of each material formulation was evaluated using the WST-8 assay (Cell Counting Kit-8, Dojindo Japan), which quantifies metabolic activity as an indicator of cell viability. Adult human dermal fibroblast cells were seeded in 96-well plates at a density of 10,000 cells per well and incubated overnight under standard culture conditions (37 °C, 5% CO_2_). Test materials were sterilized under UV light and subsequently placed in an Eppendorf tube with a cell medium ratio following the ISO-10993–5, followed by a 26 h incubation. After exposure, the incubated cellular medium was taken out, 10 μL of WST-8 reagent was added to each well containing 100 μL of culture medium, and the plates were incubated for an additional 2 h. Absorbance was recorded at 460 nm using a microplate reader (Varioskan LUX, by ThermoFisher USA). Cell viability was calculated relative to untreated control wells after blank subtraction and expressed as a percentage. In accordance with ISO 10993–5, materials were classified as noncytotoxic if cell viability was ≥70%. Each condition was tested in triplicate, and data are reported as mean ± standard deviation.

### Electrocardiography Experiment

To evaluate the performance of the L-CPE composite as a skin-interfaced electrode, electrophysiological measurements were conducted using an adult male rat. All procedures were approved by the Biological Safety and Ethics Committee of NTT Basic Research Laboratories (approval ID 2023–01), in compliance with the Guidelines for the Proper Conduct of Animal Experiments of the Science Council of Japan (Kohyo-20-k16–2, 2006). The L-CPE film was cut into a rectangular electrode patch and gently placed onto the thoracic region of the rat’s shaved skin. Commercial Ag/AgCl electrodes (NE-134A, Nihon Kohden, Japan) were used as reference and ground, positioned in adjacent regions. Electrodes were held in place using minimal adhesive tape to ensure skin contact. Electrocardiograms were recorded using a wearable device (Tx02, NTT TechnoCross, Japan), developed to monitor electrocardiograms in humans (sampling rate: 200 Hz, band-pass: 0.13 Hz–55 Hz).^61,62^ The recorded electrocardiograms were transferred to a smartphone (SH-M08, Sharp Corp., Japan) by Bluetooth Low Energy. Skin condition was visually assessed before, during, and after electrode placement to evaluate biocompatibility and dermal response.

## Supplementary Material



## Notes

Additional experimental findings, including ATR-FTIR spectra of L-CPE, M-CPE and H–CPE films; Raman spectra of pristine PEDOT:PSS, L-CPE, M-CPE,H–CPE, and PE. SEM images of CNF films at varying magnification; Relative change in sheet resistance with temperature and humidity variation for PE, L-CPE, and M-CPE; electrochemical impedance spectroscopy of PE, L-CPE and M-CPE films; beat per minutes alignment, and a scatter plot of the maximum peak amplitude of each beat; film fabrication protocol and preparation illustration; Fabrication of a patterned M-CPE electrode for on-skin applications; impedance spectroscopy of gold and M-CPE electrode on skin ; formulation table of the different volume used in preparation of the composite films; summary and comparison table of similar state of the art films (PDF)
