# Supplementary material for: Flexible Conductive Paper-Based Sensors for On-Skin Electrophysiological Monitoring and Wearable Applications
Source: ACS Appl Mater Interfaces. 2025 Dec 31;18(1):989–1002. doi: 10.1021/acsami.5c22432 (PMC12781116; doi:10.1021/acsami.5c22432)
Supplement: Supplementary file 1 [file am5c22432_si_001.pdf]

Supporting Information

Flexible conductive paper-based sensors for on-skin electrophysiological monitoring and wearable applications

George Al Boustani[1,2], Lukas Bichlmaier[2,3] , Tetsuhiko F Teshima[1,2,4,*] ,Oleksandr Berezin[5], Lennart JK Weiß[1], Koji Sakai[6], Kenji Kondo[7],Lukas Hiendlmeier[1], Defne Tüzün[1], Beatrice De Chiara[1], Marta Nikić[1], Gil G Westmeyer[5], Shigeyoshi Inoue[3] , Markus Becherer[8] , Bernhard Wolfrum[1,2,*].

1 Neuroelectronics, Munich Institute of Biomedical Engineering, Department of Electrical Engineering, TUM School of Computation, Information and Technology, Technical University of Munich, Garching 85748, Germany

2 Medical & Health Informatics Laboratories, NTT Research Incorporated, Sunnyvale, California 94085, United States

3 Chair of Silicon Chemistry, Department of Chemistry, Institute of Silicon Chemistry and Catalysis Research Center, TUM School of Natural Sciences, Technical University of Munich, 85748 Garching, Germany

4 Faculty of Science and Technology, Keio University, Yokohama, Kanagawa 223−8522, Japan

5 Neurobiological Engineering, Munich Institute of Biomedical Engineering, TUM School of Natural Sciences & TUM School of Medicine and Health, 85748 Garching, Germany

6 NTT Basic Research Laboratories and Bio-Medical Informatics Research Center, NTT Corporation, 3-1, Morinosato Wakamiya, Atsugi, Kanagawa 243-0198, Japan

7 New Materials Development Team, Wet-Jet Mill Technical Department, Plant Equipment Division, Sugino Machine Limited, 2880 Kuriyama, Namerikawa, Toyama, 936-8577, Japan

8 Chip-Based Magnetic Sensor Technology, ZEITlab TUM School of Computation, Information and Technology, Technical University of Munich, 85748 Garching, Germany

AUTHOR INFORMATION

Corresponding Author

* Dr. Tetsuhiko F. Teshima

tetsuhiko.teshima@ntt-research.com

* Prof. Dr. Bernhard Wolfrum

Bernhard.wolfrum@tum.de

Fabrication of the Wearable Device

First, the M-CPE mixture was vacuum filtered using the same method mentioned in **Figure S6-S7**. Once the composite film was completely dried, it was peeled from the PVDF membrane and transferred to a laser cutting system for patterning (**Figure S7**). Laser cutting was performed to form two set of M-CPE surface electrodes: a reference electrode with a diameter of 1 cm and a working electrode with a diameter of 0.5 cm with feedline of 2cm for both electrodes (using the following parameters: 1 W laser power, 60 kHz frequency, 1000 mm·s^{-1} scan speed, and 12 repetitions). The electrodes were attached on the surface of on-sided Kapton tape with a gap of 1.5 cm in between the two electrodes. Skin–electrode impedance spectroscopy was conducted using a PalmSens system by

applying a 100 mV AC signal over a frequency range of 1 Hz to 10 kHz. The resulting graph consist of the average and standard deviation of 3 M-CPE working electrodes and 3 same dimension 100 nm sputtered gold electrode on Polyimide films.

To evaluate the skin-electrode impedance performance of the CPE electrodes in comparison to surface gold electrodes for wearable applications, four surface electrodes and four reference electrodes were fabricated as shown in **Figure S8.** Furthermore, an impedance spectroscopy was conducted over a frequency range of 1 Hz to 10 kHz to study the skin to electrode impedance over the forehead of one participant. The M-CPE electrodes exhibited a higher impedance magnitude (|Z|) across the frequency spectrum, particularly at lower frequencies (<100 Hz), where the impedance reached an average value of $3.58 \times 10^5\ \Omega \pm 1.60 \times 10^5\ \Omega$ and $2.20 \times 10^5\ \Omega \pm 8.86 \times 10^4\ \Omega$ at 100 Hz for M-CPE and gold electrode respectively. Both gold and M-CPE showed the same impedance behavior across frequencies due to the skin impedance being he dominant factor in the measurement.

Supporting figures

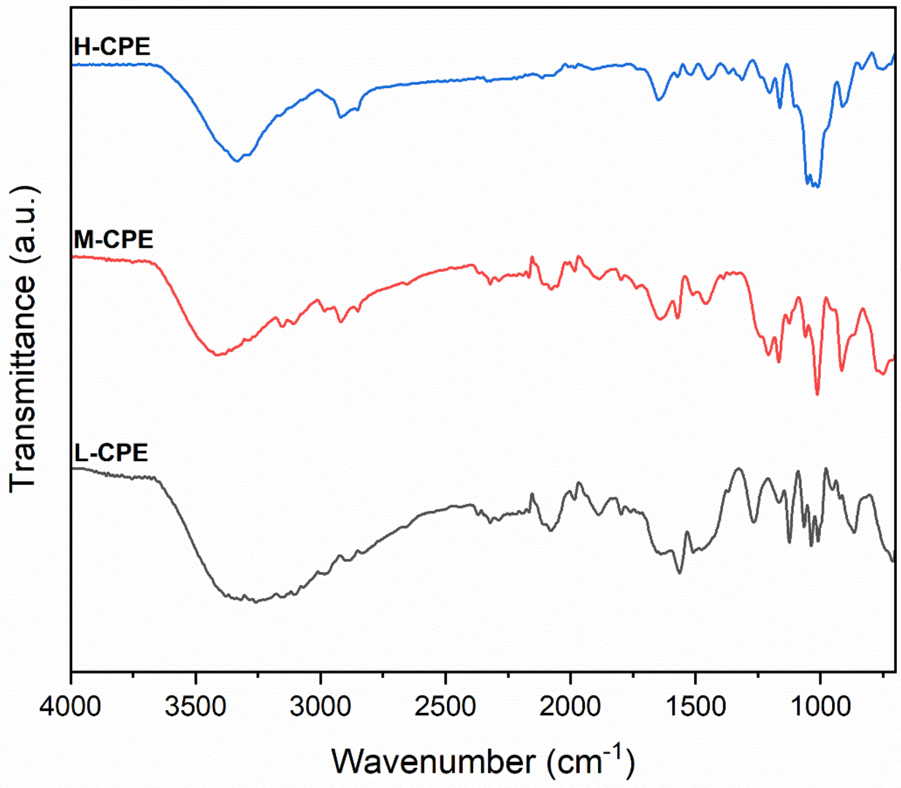

Figure S1: ATR-FTIR spectra of L-CPE, M-CPE, and H-CPE composites illustrating characteristic vibrational bands corresponding to the constituent components: cellulose, PEDOT:PSS, and EMIM ES. All spectra exhibit the broad O–H stretching vibration (~3320 cm^{-1}) from cellulose and C–H stretching from PEDOT (2950 cm^{-1} and 3160 cm^{-1}). The C=N stretching vibration (~1566 cm^{-1}) from the EMIM cation is evident, along with characteristic PEDOT bands including C=C (~1550 cm^{-1}), C-O-C (~1020 cm^{-1}) stretching. As the PEDOT and EMIM content increases from L-CPE to H-CPE, the relative intensity of the corresponding peaks also increases, confirming successful compositional variation across the series.

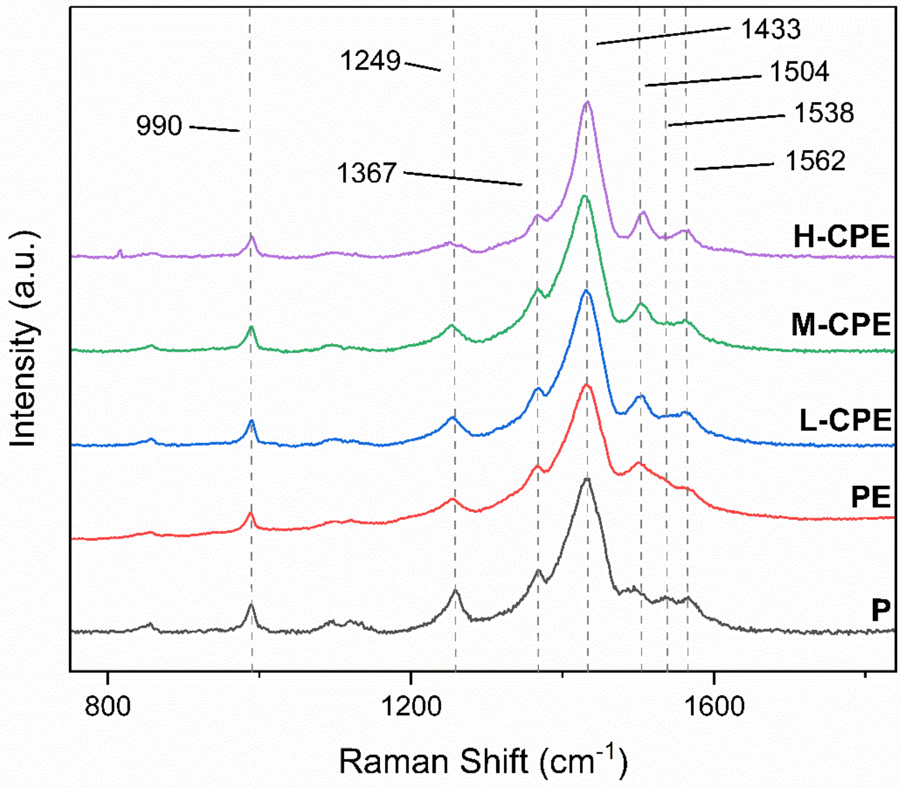

Figure S2: Raman spectra of pristine PEDOT:PSS and composite films with low (L-CPE), medium (M-CPE), high (H-CPE), and PE loadings of EMIM ES and CNF. Characteristic Raman bands at 1249, 1367, 1433, 1504, and 1538 cm^{-1} correspond to PEDOT vibrational modes, while peaks at 990 and 1562 cm^{-1} originate from the polystyrene sulfonate (PSS) component. The prominent C=C stretching mode at 1433 cm^{-1} indicates preservation of the benzoid/quinoid resonance balance in PEDOT. A gradual reduction of the ~1538 cm^{-1} band with a concomitant increase of the 1504 cm^{-1} band from PEDOT:PSS to L-CPE, M-CPE, and H-CPE suggests progressive planarization and enhanced quinoid-like conformation of the PEDOT chains with increasing EMIM ES and CNF content.

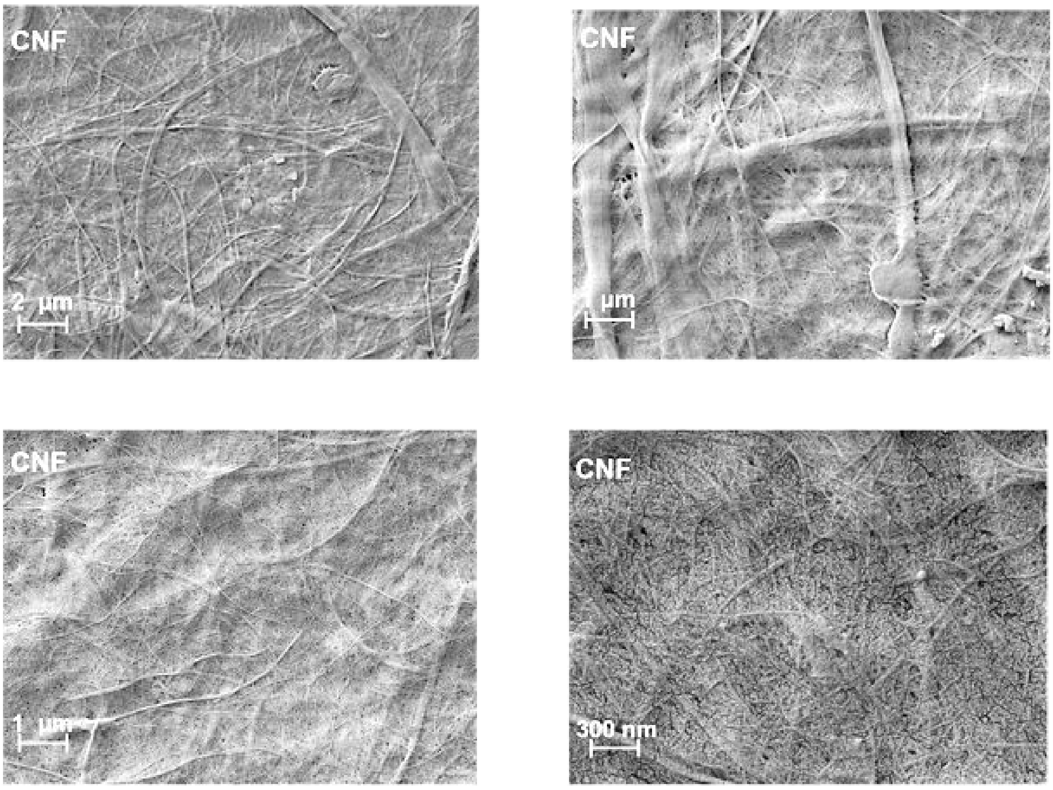

Figure S3: Scanning electron microscopy images of cellulose nanofiber films (3 nm gold coated) at varying magnifications. All images reveal a dense, interconnected network of nanofibers with randomly oriented morphologies. The micrographs show fiber entanglement and surface coverage across multiple length scales at different locations, a 2 µm image, two 1 µm images, and a 300 nm image, highlighting the nanoscale structure of the CNF network. This fibrous morphology supports mechanical reinforcement.

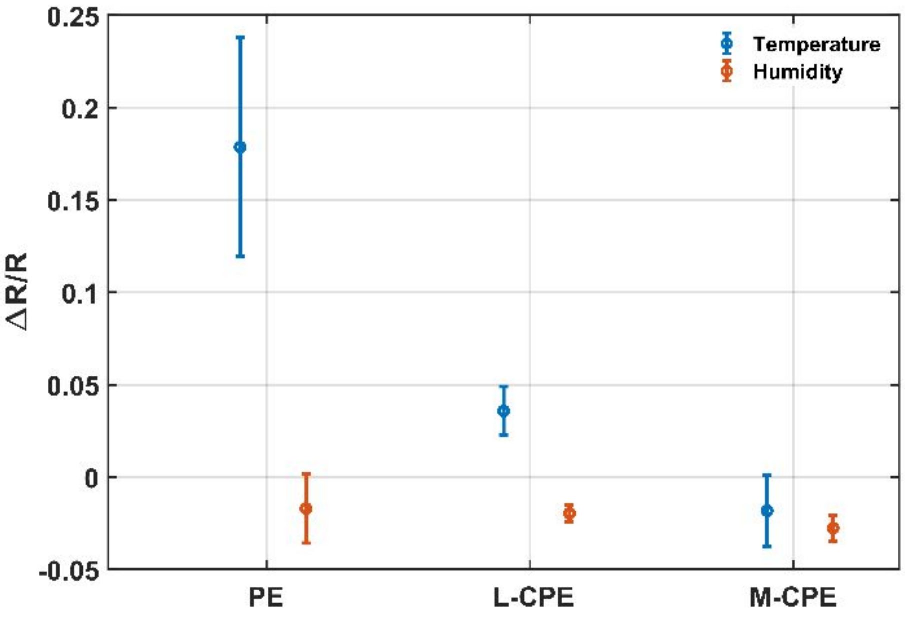

Figure S4: Relative change in sheet resistance (ΔR/R) of PE, L-CPE, and M-CPE films under temperature (blue) and humidity (orange) variations. Measurements were conducted across a temperature range of 15 °C to 55 °C at 80 %RH and a humidity range of 30%RH -90%RH at 40 °C. CNF-containing composites (L-CPE and M-CPE) exhibit significantly improved environmental stability compared to PE, with minimal variation in resistance under both thermal and moisture stress.

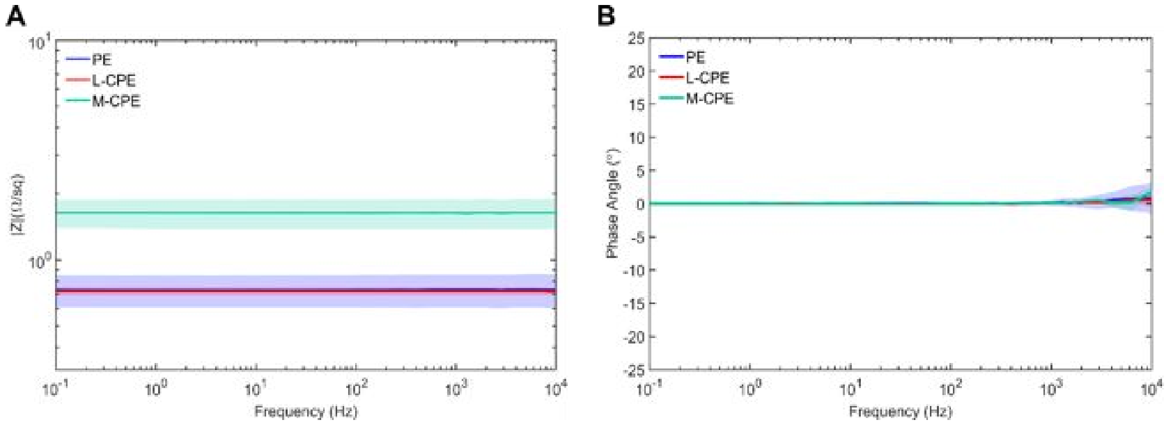

Figure S5: Electrochemical impedance spectroscopy results showing impedance magnitude (|Z|, left) and phase angle (right) as a function of frequency (0.1 Hz to 10 kHz). (A–B) Comparison of PE, L-CPE, and M-CPE electrodes under dry conditions, where all samples exhibit low and stable impedance with negligible phase shift, indicative of dominant resistive behavior. Shaded regions represent the standard deviation across three independent measurements (N = 3).

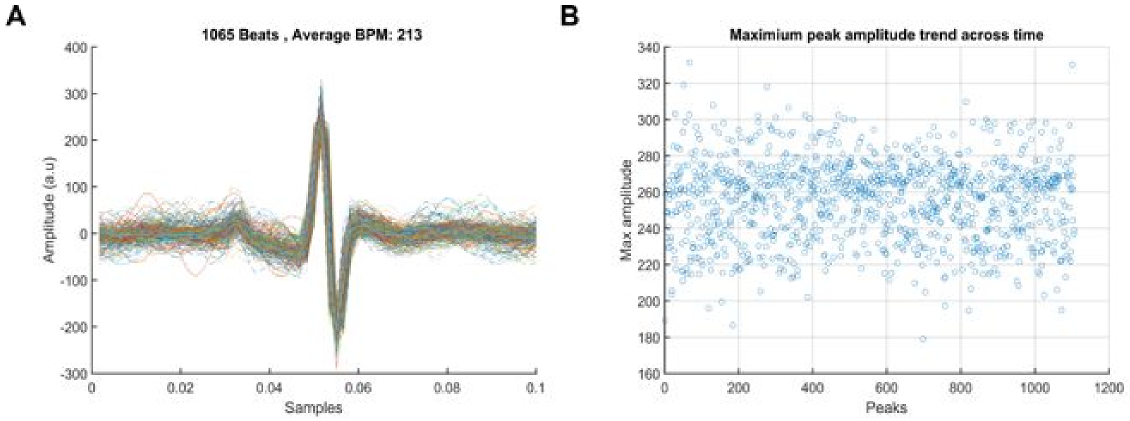

Figure S6: Beat alignment and amplitude trend analysis from in vivo electrophysiological signal acquisition using L-CPE electrodes. (A) Overlay of 1065 extracted cardiac beats showing consistent waveform morphology with an average heart rate of 213 beats per minute (BPM), demonstrating stable signal capture across time. (B) Scatter plot of the maximum peak amplitudes of each beat over time, indicating amplitude consistency with minor fluctuations. The preserved waveform shape and amplitude stability support the reliability of L-CPE electrodes for high-fidelity, long-duration physiological recordings.

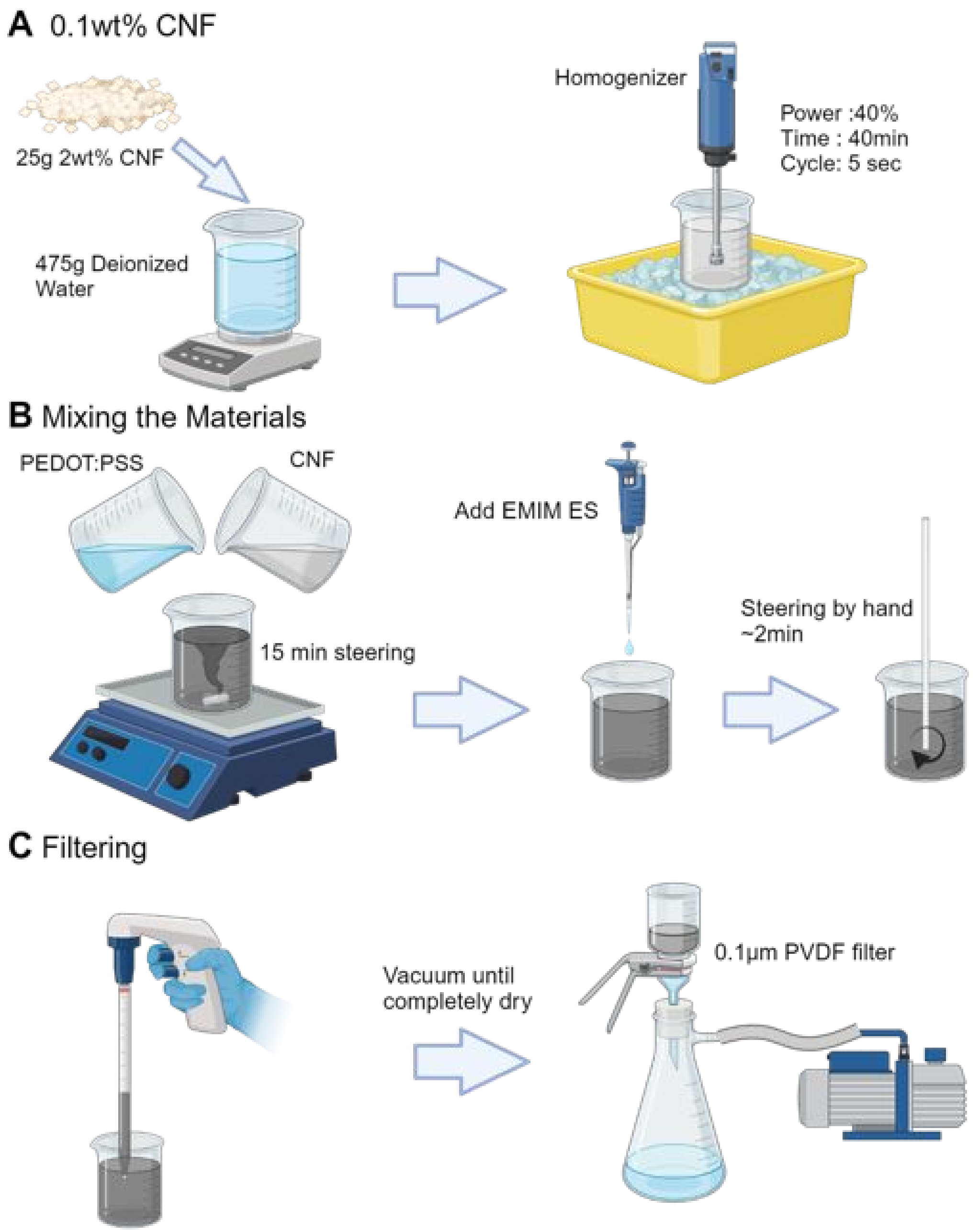

Figure S7: Schematic illustration of the solution preparation and film fabrication protocol for CNF-PEDOT:PSS-EMIM ES composites. (A) Concentrated cellulose fibers were dispersed in deionized water and subjected to high-shear homogenization in an ice bath to form a uniform cellulose nanofiber (CNF) suspension. (B) PEDOT:PSS and CNF were mixed in specific volumetric ratios. The mixture was homogenized using vortex mixing. Then, EMIM ES was pipetted to mixture and steered. (C) The resulting solution was filtered through a vacuum-assisted filtration setup to produce free-standing composite film.

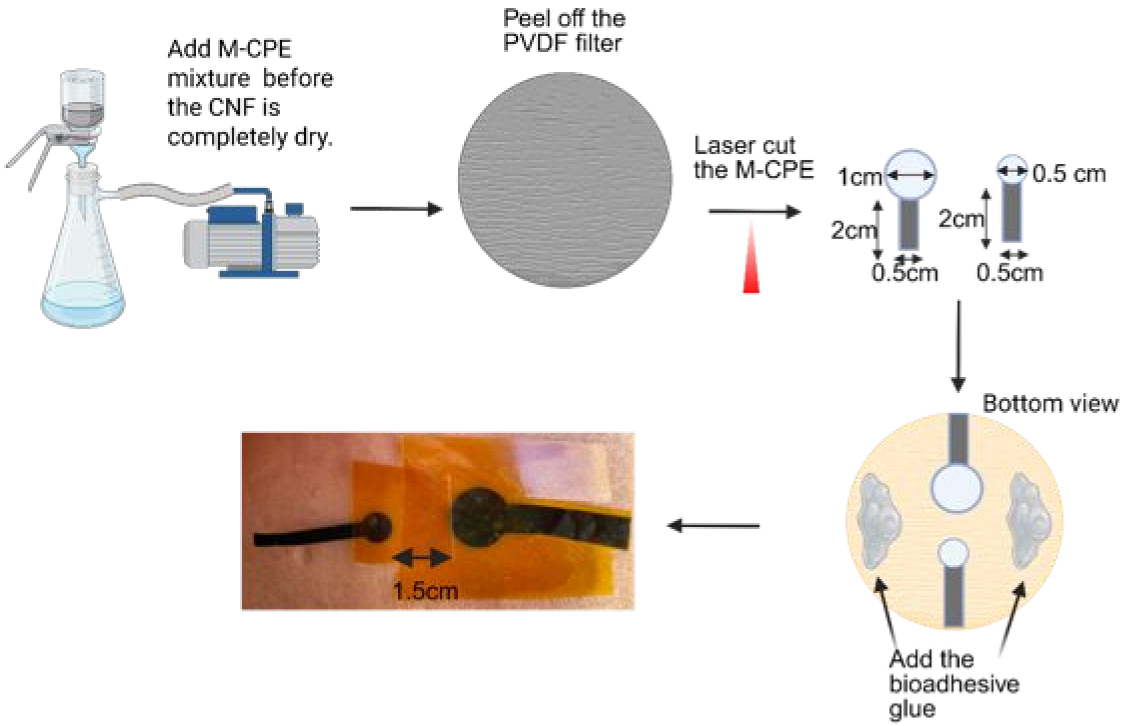

Figure S8: Schematic representation of the fabrication and integration process of patterned M-CPE/CNF electrodes. (1) A M-CPE is first formed via vacuum filtration. (2) After complete drying, the composite film is peeled off and (3) laser-cut to define two electrode areas : a reference electrode with a diameter of 1 cm and a working electrode with a diameter of 0.5 cm(4) The electrode then are attached to the surface of a one sided Kapton tape , and the mastic-gum-amylopectin adhesive was added[1]. (5) An optical image (bottom left) of the patterned electrodes confirms precise laser cutting.

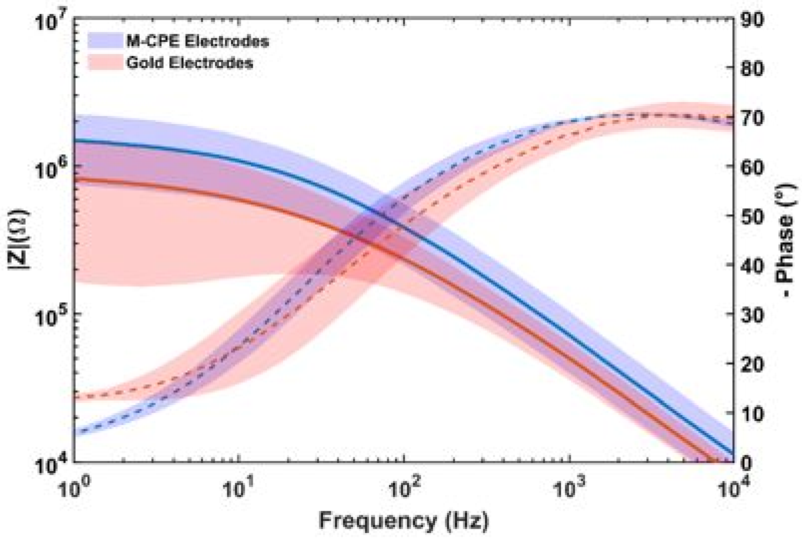

Figure S9: Bode plot comparing the impedance magnitude (|Z|, solid lines) and phase angle (–Phase, dashed lines) of M-CPE electrodes (blue) and gold electrodes (red) across a frequency range of 1 Hz to 10 kHz. Shaded regions represent standard deviation (N = 4). M-CPE electrodes exhibit higher overall impedance, particularly at lower frequencies, but maintain a comparable impedance to gold electrodes at mid- to high-frequency ranges, indicating similar capacitive behavior.

Supporting tables

Table S1: Formulation volumes of PEDOT:PSS (P), CNF (C), and 1-ethyl-3-methylimidazolium ethyl sulfate (E) used in the preparation of composite films. L-CPE, M-CPE, and H-CPE represent low-, medium-, and high-cellulose-content formulations, respectively.

Material	PEDOT:PSS (mL)	CNF (mL)	EMIM ES (mL)
L-CPE	4.09	5	0.9
M-CPE	2.27	25	2.72
H-CPE	0.45	45	4.54
PE	4.09	0	0.41
CP	4.09	5	0
CE	0	10	1

Table S2: A Summary of electrical, mechanical, and biocompatibility properties of the L-CPE, M-CPE, H-CPE composites, and similarly processed free standing films

Film	Conductivity (S cm^{-1})	Tensile Strength (MPa)	Strain at Break (%)	Mechanical Properties	Bio-compatibility
L-CPE	160	79	21	Flexible	Non-Cytotoxic, no skin Irritation, no leakage
M-CPE	40	170	24	Flexible	Non-Cytotoxic, no skin Irritation, no leakage
H-CPE	6	355	24	Flexible	Non-Cytotoxic, no skin Irritation, no leakage
PEDOT:PSS-CNF-rGO hybrid[2]	42	6	Not reported	Flexible	Not reported
PEDOT:PSS-CNF-DMSO[3]	22.6	Not reported	Not reported	Flexible	Not reported
PEDOT:PSS/PVA-DMSO	0.025	Not reported	517	Stretchable and flexible	Not reported
PEDOT:PSS-CNF-phosphoric acid[4]	3508	Not reported	3.75	Not reported	Not reported
PEDOT:PSS-CNF-DMSO[5]	66.67	72	Not reported	Flexible	Not reported
PEDOT:PSS-CNF-EG[6]	107	Not reported	Not reported	Flexible	Not reported
PEDOT:PSS-CNF-DMSO[6]	123	Not reported	Not reported	Flexible	Not reported
PEDOT:PSS-CNF[7]	252	Not reported	Not reported	Not reported	Not reported
PEDOT:PSS-BMIM octyl sulpahte[8]	525	25	30	Elastic	Not reported

References

(1) Al Boustani, G.; Xu, Z.; Teshima, T. F.; Bichlmaier, L.; Nikić, M.; Hiendlmeier, L.; Sayn-Wittgenstein, A.; Tüzün, D.; Inoue, S.; Wolfrum, B. Non-Conductive and Conductive Washable Amylopectin-Mastic Gum Adhesives for On-Skin Applications. *Advanced Materials Technologies* **2024**, *9* (24), 2400719. https://doi.org/10.1002/admt.202400719.

(2) Carrascosa, A.; Sánchez, J. S.; Morán-Aguilar, M. G.; Gabriel, G.; Vilaseca, F.; Carrascosa, A.; Sánchez, J. S.; Morán-Aguilar, M. G.; Gabriel, G.; Vilaseca, F. Advanced Flexible Wearable Electronics from Hybrid Nanocomposites Based on Cellulose Nanofibers, PEDOT:PSS and Reduced

Graphene Oxide. *Polymers* **2024**, *16* (21). https://doi.org/10.3390/polym16213035.

(3) Ko, Y.; Kim, D.; Kim, U.-J.; You, J. Vacuum-Assisted Bilayer PEDOT:PSS/Cellulose Nanofiber Composite Film for Self-Standing, Flexible, Conductive Electrodes. *Carbohydrate Polymers* **2017**, *173*, 383–391. https://doi.org/10.1016/j.carbpol.2017.05.096.

(4) Wu, X.; Huang, S.; Hu, L.; Jiang, Y.; Cui, X.; Wu, Y.; Nie, S.; Jin, Y.; Su, Z.; Song, J.; Yin, X.; Xu, Z.; Li, Z. Facile Fabrication of PEDOT:PSS-Based Free-Standing Conducting Film for Highly Efficient Electromagnetic Interference Shielding. *Macromolecular Materials and Engineering* **2023**, *308* (4), 2200554. https://doi.org/10.1002/mame.202200554.

(5) Du, H.; Zhang, M.; Liu, K.; Parit, M.; Jiang, Z.; Zhang, X.; Li, B.; Si, C. Conductive PEDOT:PSS/Cellulose Nanofibril Paper Electrodes for Flexible Supercapacitors with Superior Areal Capacitance and Cycling Stability. *Chemical Engineering Journal* **2022**, *428*, 131994. https://doi.org/10.1016/j.cej.2021.131994.

(6) Ko, Y.; Kim, J.; Kim, D.; Kwon, G.; Yamauchi, Y.; You, J.; Ko, Y.; Kim, J.; Kim, D.; Kwon, G.; Yamauchi, Y.; You, J. Fabrication of Highly Conductive Porous Cellulose/PEDOT:PSS Nanocomposite Paper via Post-Treatment. *Nanomaterials* **2019**, *9* (4). https://doi.org/10.3390/nano9040612.

(7) Chen, N.; Xie, S.; Deng, J.; Wang, B.; Yang, S.; Wang, Z. Multifunctional Highly Conductive Cellulose Nanopaper with Ordered PEDOT:PSS Alignment Enabled by External Surface Area-Promoted Phase Separation. *Composites Part B: Engineering* **2025**, *288*, 111919. https://doi.org/10.1016/j.compositesb.2024.111919.

(8) Lee, S.; Jang, J.; Lee, S.; Jung, D.; Shin, M.; Son, D.; Lee, S.; Jang, J.; Lee, S.; Jung, D.; Shin, M.; Son, D. PEDOT Composite with Ionic Liquid and Its Application to Deformable Electrochemical Transistors. *Gels* **2022**, *8* (9). https://doi.org/10.3390/gels8090534.
